# Role of long non‐coding RNAs in adipogenesis: State of the art and implications in obesity and obesity‐associated diseases

**DOI:** 10.1111/obr.13203

**Published:** 2021-01-14

**Authors:** Federica Rey, Valentina Urrata, Luisa Gilardini, Simona Bertoli, Valeria Calcaterra, Gian Vincenzo Zuccotti, Raffaella Cancello, Stephana Carelli

**Affiliations:** ^1^ Department of Biomedical and Clinical Sciences “L. Sacco” University of Milan Milan Italy; ^2^ Pediatric Clinical Research Center Fondazione “Romeo ed Enrica Invernizzi” University of Milan Milan Italy; ^3^ Obesity Unit—Laboratory of Nutrition and Obesity Research, Department of Endocrine and Metabolic Diseases IRCCS Istituto Auxologico Italiano Milan Italy; ^4^ International Center for the Assessment of Nutritional Status (ICANS), Department of Food, Environmental and Nutritional Sciences (DeFENS) University of Milan Milan Italy; ^5^ Pediatrics and Adolescentology Unit, Department of Internal Medicine University of Pavia Pavia Italy; ^6^ Department of Pediatrics Children's Hospital “V. Buzzi” Milan Italy

**Keywords:** adipogenesis, lncRNAs, metabolic diseases, obesity

## Abstract

Obesity is an evolutionary, chronic, and relapsing disease that consists of a pathological accumulation of adipose tissue able to increase morbidity for high blood pressure, type 2 diabetes, metabolic syndrome, and obstructive sleep apnea in adults, children, and adolescents. Despite intense research over the last 20 years, obesity remains today a disease with a complex and multifactorial etiology. Recently, long non‐coding RNAs (lncRNAs) are emerging as interesting new regulators as different lncRNAs have been found to play a role in early and late phases of adipogenesis and to be implicated in obesity‐associated complications onset. In this review, we discuss the most recent advances on the role of lncRNAs in adipocyte biology and in obesity‐associated complications. Indeed, more and more researchers are focusing on investigating the underlying roles that these molecular modulators could play. Even if a significant number of evidence is correlation‐based, with lncRNAs being differentially expressed in a specific disease, recent works are now focused on deeply analyzing how lncRNAs can effectively modulate the disease pathogenesis onset and progression. LncRNAs possibly represent new molecular markers useful in the future for both the early diagnosis and a prompt clinical management of patients with obesity.

**Abbreviations**
ADINR: adipogenic differentiation‐induced ncRNA
AdipoQ AS: adiponectin antisense RNA
ADNCR: adipocyte differentiation‐associated lncRNA
AF: atrial fibrillation
AngII: angiotensin II
ANRIL: antisense ncRNA in the INK4 Locus
APF: autophagy promoting factor
ASMER‐1 and ASMER‐2: adipocyte‐specific metabolic‐related lncRNAs
BMI: body mass index
CAIF: cardiac autophagy inhibitory factor
CARL: cardiac apoptosis‐related lncRNA
CDKN2B‐AS1: cyclin‐dependent kinase inhibitor 2B antisense RNA 1
Chaer: cardiac‐hypertrophy‐associated epigenetic regulator
CHD: coronary heart diseases
CHRF: cardiac hypertrophy‐related factor
CIDEC: cell death‐inducing DFF45‐like effector
CVD: cardiovascular diseases
DGAT: diacylgycerolacyltransferase
DN: diabetic nephropathy
DNMT1: DNA methyl transferase 1
Giver: Growth factor‐ and pro‐Inflammatory cytokine‐induced Vascular cell‐Expressed lncRNA
hADSCs: human adipose‐derived stem cells
HFD: high‐fat diet
hLMR: human lncRNA metabolic regulators
HOTAIR: HOX transcript antisense RNA
HRCR: heart‐related circRNA
IMFNCR: intramuscular fat‐associated lncRNA
IR: insulin resistance
LIPCAR: long intergenic non‐coding RNA predicting cardiac remodeling
lnc‐ORA: obesity‐related lncRNA
lncRNA: long non‐coding RNAs
MALAT1: metastasis‐associated lung adenocarcinoma transcript 1
MCE: mitotic clonal expansion phase
MDRL: mitochondrial dynamic‐related lncRNA
Meg3: maternally expressed gene 3
MHRT: myosin heavy chain associated RNA transcripts
MIAT: myocardial infarction‐associated transcript
MIR221HG: miR‐221 host gene
MIR31HG: miR‐31 host gene
MIRT1: myocardial infarction‐associated transcript 1
NAFLD: nonalcoholic fatty liver disease
OA: osteoarthritis
PRC2: Polycomb Repressor Complex 2
SD: standard deviations
SRA: steroid receptor RNA activator
T2D: type 2 diabetes
TINCR: tissue differentiation‐inducing non‐protein coding RNA
VSMCs: vascular smooth muscle cells
WHO: World Health Organization
Wisper: Wisp2 super‐enhancer‐associated RNA
βlinc1: β‐cell long intergenic noncoding RNA

## 1 INTRODUCTION TO OBESITY: CAUSES AND CONSEQUENCES

Obesity is defined by the World Health Organization (WHO) as a condition of abnormal or excessive accumulation of body fat that presents a health risk, increasing both morbidity (for many chronic diseases such as type 2 diabetes [T2D], hypertension, coronary artery disease, dyslipidemia, stroke, osteoarthritis (OA), and even certain forms of cancer)^1^, ^2^, ^3^, ^4^, ^5^ and mortality.^3^ The most recent report of the WHO shows how the worldwide prevalence of obesity nearly tripled between 1975 and 2016, as over 650 million adults clinically were affected by obesity and 41 million children below the age of 5 and over 340 million children and adolescents between 5 and 19 years of age were either overweight or affected by obesity.^3^, ^6^ Indeed, studies show that 70% of adolescents with obesity will maintain their obese condition as adults, with a significant impact on their physical and physiological health.^3^, ^7^, ^8^, ^9^ Specifically, an adult is affected by obesity when his/her body mass index (BMI) is greater than or equal to 30.^3^ In the pediatric age, according to the WHO, obesity in children under 5 years of age is defined as weight‐for‐height 3 standard deviations (SD) above the WHO Child Growth Standards reference median. For children aged 5–19 years, obesity is defined as BMI‐for‐age 2 SD above the WHO Growth Standards reference median.^6^

Conventional therapies for patients with obesity, such as lifestyle modifications (diet and exercise) and also pharmacotherapy in adults, remain important but are limited by their results in terms of weight loss and weight loss maintenance at long term, and in the next future the development of new combinatory clinical approaches is needed.^10^, ^11^, ^12^, ^13^ From a cellular perspective, obesity is caused by the excessive accumulation of adipose cells in different anatomical parts of the body. This is due to an increase in adipocytes' size (hypertrophy), number (hyperplasia) both and even in an imbalance of the adipogenesis process.^14^, ^15^ At present, it remains thus necessary to continue research on the biological basis of this complex pathology starting from genetic, epigenetic, and molecular pathways as it is not possible to conclude what the relative contribution of genetics and environment are in obesity onset. Indeed, behavior and genes are different levels of the same causal framework, and epigenetics through RNA biology might play a central role in elucidating new targetable pathways. “Classic” epigenetic mechanisms and epigenetic mosaicism, a widespread phenomenon documented in many organisms, that may account for differences in body weight and fat accumulation among people remain to be better investigated,^16^, ^17^, ^18^ taking into account of the role of non‐coding RNAs as possible epigenetic modulator of obesity and secondary co‐morbidities onset. In this review, we aim to discuss the functional roles of long non‐coding RNAs (lncRNAs), focusing on the state of the art and the future clinical implications of lncRNAs in adipogenesis, obesity, and obesity complications onset.

## 2 LNCRNAS: DEFINITION AND PRINCIPAL FUNCTIONS

In recent years, the role of RNA is changed, and indeed it is now established knowledge that only 1–2% of the human genome codes for protein.^19^, ^20^, ^21^ For this reason, RNAs can be classified for their coding potential in coding RNAs (transcripts that will subsequently be translated into proteins) and non‐coding RNAs that do not code for a polypeptide and whose function is still to be fully understood especially in modulating gene expression.^19^, ^20^, ^21^ Among the non‐coding RNAs, it is possible to distinguish two subclasses: small non‐coding RNAs, molecules smaller than 200 bp, and lncRNAs, defined as non‐coding RNA molecules longer than 200 bp.^22^ LncRNAs are poorly conserved, frequently unstable, and/or sometimes present in few copies, and new biological roles have emerged for some lncRNAs.^23^, ^24^, ^25^ In order to facilitate the reader through this mounting evidence in different models, the lncRNAs reported in this work are listed for their homology as summarized in Table S1.

Interestingly, lncRNAs can mediate transcriptional regulation in different ways. Indeed, these molecules can modulate gene expression at multiple levels, ranging from chromatin re‐arrangements to transcriptional regulation or even translational modulation.^26^, ^27^, ^28^ Multiple pieces of evidence suggest that they can operate through distinct modes, including working as signals, scaffolds for protein–protein interactions, molecular decoys, and guides to target elements in the genome or transcriptome.^29^ This high degree of complexity in gene expression regulation, and the number of still unknown mechanisms through which lncRNAs could act, indicates a clear need to further investigate these molecules, both in health and disease, as they could provide crucial new insights in cell biology representing promising targets for the development of innovative therapeutic strategies for multiple diseases, with a specific relevance for their epigenetic regulation of metabolic diseases. Indeed, the non‐coding transcriptome is becoming more and more relevant also in the field of adipogenesis, fat mass expansion, and obesity, and in this context lncRNAs represent new potential candidate targets for the development of therapies.^23^, ^24^, ^25^

## 3 LNCRNAS IN ADIPOGENESIS AND OBESITY

Noncoding RNAs are known to play a regulatory role in many developmental contexts, including adipogenesis. Indeed, lncRNAs have been demonstrated to be involved in adipogenesis with subsequent implications for obesity and obesity‐related complications in adults and children.^30^, ^31^, ^32^ As more and more studies in this field arise every year, there is a need to distinguish between the multiple functions that the lncRNAs could have. Indeed, results are variable, and a full characterization of the role that lncRNAs play in obesity is far from being present. Numerous lncRNAs have been correlated with adipogenesis, and the aim of this section is thus to classify them accordingly to their role in different stages of adipocytes differentiation, subsequently focusing on their role in obesity.

### 3.1 Role of lncRNAs in the regulation of early adipogenesis master regulators

Adipogenesis is the process of adipocytes formation into fat‐containing cells from stem cells or adipocyte precursors. It involves two phases: determination (considered an early stage) and terminal differentiation (late adipogenesis).^14^, ^33^, ^34^

Early stages of adipogenesis are represented by a mitotic clonal expansion phase (MCE) and by the expression of early regulators such as C/EBPβ and C/EBPδ.^34^, ^35^, ^36^ Among the lncRNAs able to influence this stage of adipogenesis, the lncRNA steroid receptor RNA activator (SRA) was one of the first to be described.^37^ Its expression resulted twofolds higher in differentiated murine 3T3‐L1 adipocytes than pre‐adipocytes, but the lncRNA seems to also act in early phases of adipogenesis.^38^ Indeed, it can promote S‐phase entry during the MCE of adipogenesis controlling cell cycle genes' expression.^37^ Moreover, in the mouse ST2 mesenchymal cell line, SRA is implicated in the regulation of p38/JNK′ phosphorylation inhibition, a crucial step in the early stages of adipogenesis, as well as in stimulating insulin receptor gene expression and downstream signaling.^39^, ^40^ The obesity‐related lncRNA (lnc‐ORA), whose expression levels increases during adipogenesis in obese mice, also regulates the cell cycle through induction of expression of crucial marker genes such as PCNA, cyclin B, cyclin D1, and cyclin E.^41^ Modulation of the cell cycle and thus early stages of adipogenesis can also occur through epigenetic modulation, and indeed the lncRNA slincRAD was found to interact with the DNA methyl transferase 1 (DNMT1) in the S phase of the cell cycle in mouse, facilitating the cell's entry into the MCE phase.^42^ Through microarray study a novel lncRNA, the lncRNA‐Adi, has been identified and found to be highly expressed in the MCE phase in rat adipocytes. It exerts its effect through the interaction with miR‐449a, enhancing the expression of the miRNA's target CDK6, a cyclin‐dependent kinase sensitive to high‐fat diet (HFD) and involved in the regulation of cell beige tissue formation.^43^, ^44^

The genetic location of lncRNAs could be of crucial relevance in identifying their target genes. Three recently discovered lncRNAs, Gm15051, Tmem189, and Cebpd genomically, locate respectively next to Hoxa1, C/EBPβ, and C/EBPδ in mouse, and their expression levels correlate, suggesting that each of them can positively influence the neighboring gene's expression having the role of transcriptional regulators.^45^

### 3.2 Role of lncRNAs in the regulation of late adipogenesis master regulators

As pre‐adipocytes mature into adipocytes, C/EBPβ and δ target the promoters of C/EBPα and PPARγ, master regulators of adipocytes terminal differentiation as they activate genes that are involved in insulin sensitivity, lipogenesis, and lipolysis, with subsequent implications for diseases involving lipid metabolism such as dyslipidemia.

**FIGURE 1:**
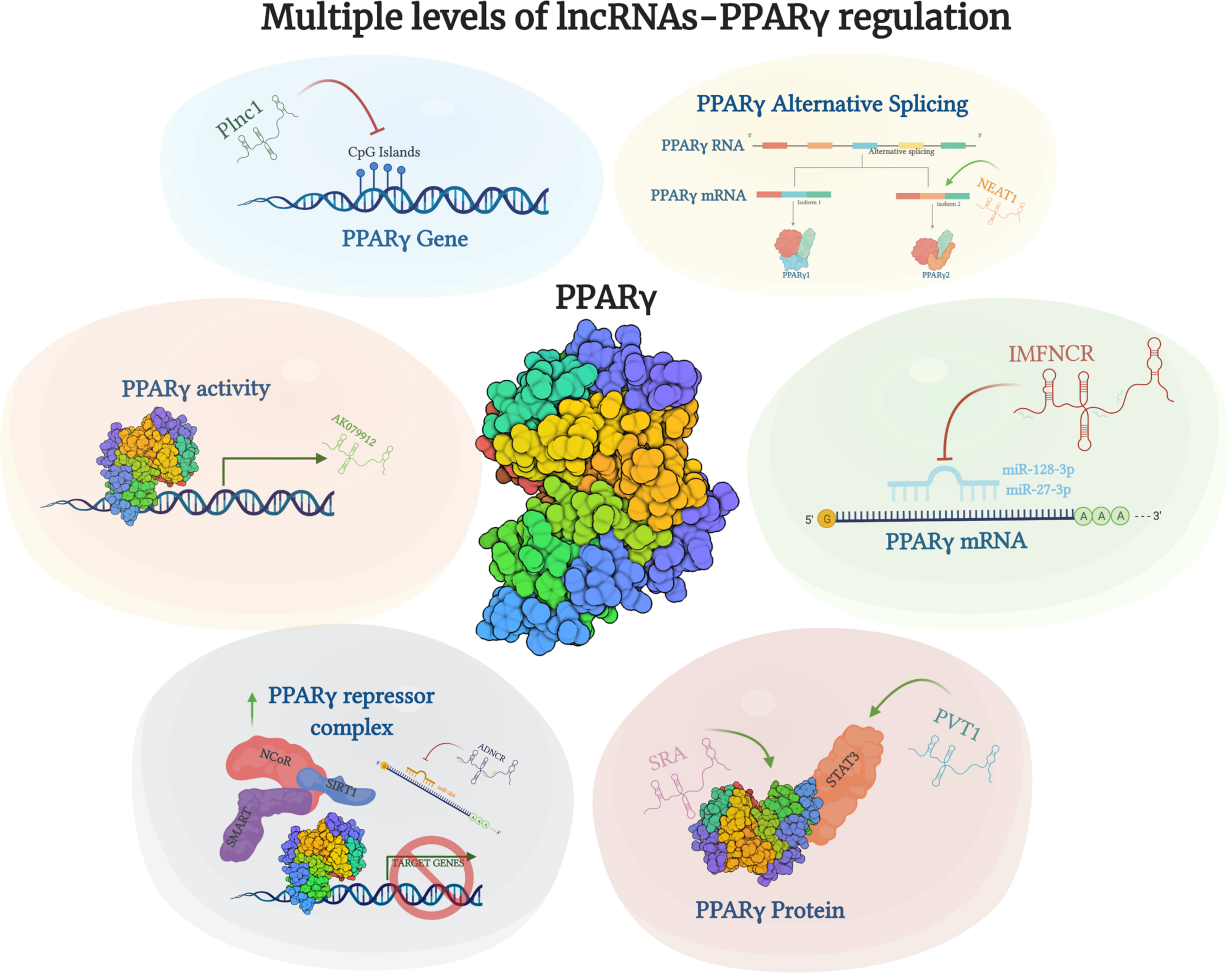
LncRNAs can influence PPARγ's transcription and activity at multiple levels. Specifically, lncRNAs can modulate directly PPARγ by inhibiting DNA methylation. They can also selectively induce a different PPARγ mRNA splicing or sponge‐specific miRNAs which would sequester and lead to degradation of PPARγ's mRNA. They directly bind to the PPARγ protein being able to inhibit its activity through upregulation of the PPARγ repressor complex. Lastly, PPARγ itself can induce the expression of specific lncRNAs. Made in ©BioRender—biorender.com

This step is critical for late adipocyte differentiation, and indeed numerous lncRNAs have been found to modulate specifically PPARγ (Figure 1), along with other late‐adipogenesis regulators. SRA also plays a role in this context, as it exerts its function via direct association with the PPARγ protein in murine cells, promoting its transcriptional activity.^37^, ^38^ Another mode of action through which lncRNAs can modulate PPARγ is through miRNA sponging. This is the case of lncRNA IMFNCR (intramuscular fat‐associated lncRNA), which has been found to promote intramuscular adipocyte differentiation in chicken sponging miR‐128‐3p and miR‐27b‐3p, which directly target PPARγ.^46^ There can also be an indirect lncRNA‐miRNA modulation of PPARγ, through other epigenetic modulators. The adipocyte differentiation‐associated lncRNA (ADNCR) can sponge miR‐204, whose target gene, SIRT1, is known to form a complex with modulators such as NCoR and SMART to repress PPARγ activity in bovine adipocytes.^47^ An epigenetic modulation can happen at PPARγ's promoter, in sites known as CpG islands that when methylated decrease the expression of the respective downstream genes. Indeed, the lncRNA Plnc1, transcribed 25,000 bp upstream of PPARγ2, can attenuate the methylation status of its promoter increasing subsequent transcription in mouse.^48^ PPARγ can also be targeted at the end of specific signal transduction pathways, as demonstrated for STAT3 gene expression regulation.^49^ Specifically, adipogenesis is induced by the activation of STAT3, acting as a molecular switch. This effect was counteracted by PPARγ's activation with the agonist troglitazone, suggesting that STAT3 can modulated adipogenic differentiation through a PPARγ upstream regulation.^49^ The nuclear lncRNA PVT1 has been found to associate with STAT3 in 3T3‐L1 pre‐adipocytes, and indeed PVT1 has been found to correlate with increased expression of PPARγ, but also C/EBPα, FABP4, and genes related to fatty acid synthesis.^50^ Well‐renowned lncRNAs, such as NEAT1, widely implicated in numerous cancers, can also have a function in adipogenesis, and indeed NEAT1 has been found to modulate the splicing of PPARγ, increasing the expression of the isoform 2 through SRp40 association in 3T3‐L1 pre‐adipocyte.^51^ PPARγ can itself regulate lncRNA's expression, such as AK079912, which presents three conserved PPARγ binding sites in its promoter region^52^ or lnc‐BATE in mouse.^53^

PPARγ is not the only player in late adipogenesis, and indeed, lncRNAs can modulate other key targets. Specifically, knockdown of the lncRNA HOXA11‐AS1 can result in the inhibition of adipocyte differentiation through a decrease of C/EBPα, diacylgycerolacyltransferase (DGAT) 2, cell death‐inducing DFF45‐like effector (CIDEC), and perilipin.^54^ On the other hand, the tissue differentiation‐inducing non‐protein coding RNA (TINCR) can form a feedback loop with miR‐31 and C/EBPα, promoting adipogenesis in human adipose‐derived stem cells (hADSCs).^55^ The adipogenic differentiation‐induced ncRNA (ADINR) can activate MLL3/4, epigenetically modulating transcription of C/EBPα in hADSCs.^57^, ^58^ LncRNAs can also bind epigenetic regulators and upregulate expression of late‐adipogenesis genes, as does miR‐31 host gene (MIR31HG), which is able to promote the binding of H3K4me3 to FABP4's promoter, increasing its expression in hADSCs.^56^ The Wnt/β‐catenin signaling is also influenced by a novel nuclear lncRNA, AC092834.1 in hADSCs. This lncRNA directly promoted an increase in the expression of DKK1, which competitively binds to LRP5 to degrade cytosolic β‐catenin, ultimately leading to upregulation of adipogenic transcripts such as PPARγ, FAPB4, and C/EBPα.^57^

A specific subclass of lncRNAs, defined as “antisense RNAs,” can modulate the expression of their respective sense gene altering processes in which they are involved. For example, PU.1AS can form a RNA‐duplex with PU1, a molecule that inhibits adipogenesis, hindering its expression and subsequent protein expression with a decreased expression of PPARγ, fatty acid synthase, and adiponectin in mouse.^58^, ^59^ Similarly, adiponectin antisense RNA (AdipoQ AS) can modulate adiponectin expression and inhibit murine adipogenesis.^60^ Although not its antisense, lnc‐leptin is directly correlated with leptin, as it is transcribed from an enhancer region upstream of leptin and their expression directly correlates.^61^

The lncRNA's correlation with adipogenesis can also be negative, as some lncRNAs have been found to be decreased in adipogenesis, such as lnc‐U90926 in murine 3T3‐L1 pre‐adipocytes,^62^ miR‐221 host gene (MIR221HG) in bovine adipocytes, and lncRNA H19 in human bone marrow mesenchymal stem cells.^63^, ^64^

Further studies might be needed to clarify specific lncRNA's functions in this process, as controversial evidences are also present. This is the case of maternally expressed gene 3 (Meg3), a novel lncRNA which has been defined as both able to inhibit and promote adipogenesis.^65^, ^66^ Indeed, a first study reported that silencing of Meg3 promoted adipogenesis through the overexpression of the adipogenesis‐related miR‐140‐5p, PPARγ, and C/EBPα, suggesting that when Meg3 is absent, adipogenesis is induced.^65^ On the contrary, another work described Meg3's role in upregulating Dickkpof‐3 through interaction with miR‐217, ultimately leading to an upregulation of adipogenesis via the induction of expression of adipogenesis‐related genes such as FABP4.^66^ This might be due to a time‐specific effect of the lncRNA's action, or the different cellular context as the first study was performed in human cells whereas the second in murine 3T3‐L1 pre‐adipocytes.

### 3.3 Identification of lncRNAs specifically associated with obesity

Specific studies correlate lncRNAs with the obese phenotype and obesogenic models. Among them, SRA has been demonstrated to be strictly associated with obesity, as it has been shown that SRA^−/−^ mice have a phenotype of resistance to HFD‐induced obesity with decreased fat mass, reduced fatty liver, and improved glucose tolerance.^67^ High‐throughput techniques such as RNA sequencing allowed the screening of the whole transcriptome in adipose tissue of patients with obesity versus lean individuals, leading to the identification of novel lncRNAs involved in the disease. In one study, two lncRNAs termed adipocyte‐specific metabolic‐related lncRNAs (ASMER‐1 and ASMER‐2) were identified and found to regulate adipogenesis, lipid mobilization, and adiponectin secretion.^68^ Screenings were also performed in gluteal subcutaneous adipose tissue on healthy subjects, in which 120 adipose‐derived lncRNAs were identified^69^ and in children with obesity, with the identification of 1268 lncRNAs, and a specific relevance for RP11‐20G13.3 has been found.^31^ The same has been done in mice, where brown and white adipocytes, pre‐adipocytes, and cultured adipocytes were screened leading to the identification of 175 different lncRNAs that are specifically regulated during adipogenesis in one study^70^ and 735 upregulated and 877 downregulated lncRNAs in murine brown versus white adipocytes.^71^ Similarly, inguinal white adipose tissue has been screened in obese mice compared to wild type ones, identifying 46 differentially expressed lncRNAs.^41^ Moreover, lncRNAs such as PVT1 and Plnc1 were found to be upregulated in obese mice.^48^, ^50^

From an anatomical point of view, lncRNAs expression can differ in different fat depots, as it is for HOX transcript antisense RNA (HOTAIR) which has been demonstrated to be highly expressed in gluteal‐femoral fat, and mechanical stimulation of this area in human subjects induces exosomal secretion of HOTAIR, which then circulates in the bloodstream resulting in higher serum expression in subjects with obesity and a sedentary lifestyle.^72^

## 4 LNCRNAS IN OBESITY‐ASSOCIATED DISEASES

Given the strong implications of lncRNAs in adipogenesis and adipocytes differentiation, it was a natural evolution to study the role of these molecular modulators in obesity and in the related most common complications.^73^, ^74^, ^75^ The obesity‐associated diseases are numerous,^1^, ^2^, ^3^ and the initiating events start early in childhood.^76^ Indeed, very recently numerous lncRNAs have been found to correlate with obesity‐associated inflammatory diseases.^77^ The following sections summarize recent advances in identifying lncRNAs implicated in cardiovascular complications (such as myocardial infarction, coronary heart diseases (CHD), cardiac hypertrophy, heart failure, atrial fibrillation (AF), and atherosclerotic thrombosis), endocrine/metabolic complications (such as T2D and nephropathy), and even immune‐related complications (such as OA) which are obesity‐associated and/or regulated.

### 4.1 Cardiovascular diseases

Cardiovascular diseases (CVD) include myocardial infarction, CHD, cardiac hypertrophy, heart failure, AF, and atherosclerotic thrombosis.^73^, ^78^, ^79^, ^80^ Childhood and adolescent obesities play a crucial role in developing CVD risk factors and are linked to higher risk of cardiovascular morbidity and mortality in adulthood.^81^ Numerous lncRNAs are implicated in CVD, and among them cardiac autophagy inhibitory factor (CAIF) is downregulated in end‐stage cardiomyopathy and usually could represent a good biomarker of a disease state in humans.^82^ CAIF seems to have a protective role through suppression of cardiac autophagy while directly blocking p53. P53 is known to target and upregulate myocardin in myocardial ischemia and reperfusion, and CAIF thus indirectly inhibited myocardin's expression.^83^ It has been reported that antisense ncRNA in the INK4 Locus (ANRIL) can sponge miR‐99a and miR‐449 during autophagy processes, subsequently upregulating thrombomodulin and promoting angiogenesis in human umbilical vein endothelial cells.^84^ The lncRNA autophagy promoting factor (APF) can also influence autophagic cell death in murine myocardial infarction targeting miR‐188‐3p and autophagy‐related protein 7.^85^ A third lncRNA which can modulate murine autophagy through miRNA sponging is AK088388, regulating Beclin‐1 and LC3‐II's expression through miR‐30a.^86^

LncRNAs can also target the apoptotic process in cardiomyocytes, which can lead to myocardial infarction. P53 is also implicated in apoptosis modulation, and the lncRNA Meg3 can target p53 and subsequently modulate NF‐κB‐ and ERS‐associated apoptosis in murine ventricular myocytes.^87^ Cardiac apoptosis‐related lncRNA (CARL) is able to sponge miR‐539 in mice and thus indirectly upregulate its target PHB2, which modulates apoptosis and mitochondrial fission.^88^ Mitochondrial fission and fusion are indeed strictly associated with cardiomyocyte apoptosis. The lncRNA AK009271, named mitochondrial dynamic‐related lncRNA (MDRL), has been proved to be involved in mitochondrial fission and fusion under stress conditions. MDRL can interact with miR‐361 and suppress it, thus reducing mitochondrial fission and apoptosis upon anoxia/reoxygenation treatment in murine cardiomyocytes.^89^, ^90^ A specific analysis of lncRNAs involved in myocardial infarction has been performed by Chen and colleagues, which reports numerous studies aimed at performing high‐throughput screening of lncRNAs which are differentially expressed in various heart diseases.^91^ They also report an implication for the lncRNAs ZFAS1,^92^, ^93^ HOTAIR,^94^ MALAT1,^95^, ^96^ GAS5,^97^ FAF,^98^ TTTY15,^99^ ECRAR,^100^ AK080084,^101^ NR_045363,^102^ TUG1,^103^ and Meg3.^91^, ^104^

Myocardial infarction can indeed influence a differential lncRNAs expression. Specifically, acute myocardial infarction in mice was associated with the upregulation of two lncRNAs named myocardial infarction‐associated transcript 1 (MIRT1) and 2 (MIRT2), which negatively correlated with infarct size and positively correlated with ejection fraction. MIRT1 and MIRT2 modulate the expression of multiple genes known to be involved in processes affecting left ventricular remodeling, such as extracellular matrix turnover, inflammation, fibrosis, and apoptosis.^105^ The lncRNA metastasis‐associated lung adenocarcinoma transcript 1 (MALAT1) has been seen expressed in cardiomyocytes subjected to hypoxia, high glucose, cytokine, and oxidative stress which are all risk factors of CVD in human and murine models, and thus has been suggested to represent a new possible therapeutic target in the disease.^106^ The lncRNA myosin heavy chain associated RNA transcripts (MHRT) was upregulated in blood of patients with myocardial infarction and seems to be upregulated in cardiac myocytes in the presence of high levels of reactive oxygen species to exert protective effect on these cells.^107^ The lncRNA Wisp2 super‐enhancer‐associated RNA (Wisper) was induced in cardiac fibrosis in both human patients and murine models, where it could be protective through regulation of cardiac fibroblast proliferation, migration, and survival.^108^ MIAT has been found to be upregulated in serum of patients with coronary atherosclerotic heart disease.^109^ MIAT can also sponge and thus inhibit miR‐133a‐3p, protective in multiple heart diseases for its role in improving cardiac function and decreasing fibrosis in rat models.^110^

LncRNAs can also influence cardiac hypertrophy and thus aggravate CVD, as cardiac hypertrophy is a crucial hallmark of heart failure.^111^ Indeed, the heart‐enriched lncRNA cardiac‐hypertrophy‐associated epigenetic regulator (Chaer), can epigenetically interact with the Polycomb Repressor Complex 2 (PRC2) and inhibit histone H3 lysine 27 methylation at the promoter regions of genes involved in cardiac hypertrophy, thus inducing the expression of genes involved in cardiac hypertrophy, with studies performed in rat, murine, and human cells.^112^ Cardiac hypertrophy can also be induced by the lncRNA cardiac hypertrophy‐related factor (CHRF) in mouse, although in this case the underlying mechanisms involves sponging of miR‐489 and subsequent upregulation of the miRNA's target Myd88, a regulator of cardiomyocyte hypertrophy.^113^ The lncR‐UCA1 is upregulated in mice hypertrophic cardiomyocytes, and it can sponge miR‐184, enhancing the expression of HOXA9.^114^ A detailed report on lncRNAs in cardiac hypertrophy is reported in the work by Liu and colleagues,^115^ which also implicates the lncRNAs MHRT,^116^ Meg3,^117^ DACH1,^118^ H19,^119^ Plscr4,^120^ SNHG1,^121^ TINCR,^122^ Uc.323,^123^ and Ahit.^124^ Other lncRNAs have also been implicated in heart failure,^111^ as does the heart‐related circRNA (HRCR), which in mice was found to acts as endogenous sponge to mir‐223, protecting them from hypertrophic stimuli.^125^ Moreover, the lncRNA HypERlnc was significantly reduced in human cardiac tissue from patients with heart failure compared with controls.^126^ Moreover, the lncRNAs profile was analyzed in plasma of patients with ischemic cardiomyopathy and dilated cardiomyopathy, two major problems which lead to heart failure.^127^ This microarray analysis identified 3222 differentially expressed lncRNAs, highlighting also a co‐expression between lncRNAs and mRNAs.^127^ Other high‐throughput screening for lncRNAs in heart failure were performed in rat models of ischemic heart failure,^128^ in murine models of post‐myocardial infarction,^129^ in explanted human heart failure hearts versus control donated ones,^130^ and in left ventricle biopsies of patients affected by non‐end‐stage dilated ischemic cardiomyopathy and matched controls^131^ highlighting a substantial number of lncRNAs implicated in the pathophysiology of this process.^91^, ^132^, ^133^

Another form of CVD is AF, which is the most common type of arrhythmia.^134^ Numerous studies were performed on the role of lncRNAs in this disease, and also in this case high‐throughput screening has allowed the identification of mounting evidences on lncRNAs in this disease.^135^ Specifically, a study conducted in right atrium tissue of patients with rheumatic heart diseases and AF or normal sinus rhythm highlighted 182 differentially expressed lncRNAs.^134^ Another work identified the transcriptome profile of left and right atrial appendages of patients with AF versus controls and identified NPPA and its antisense as potential regulators of muscle contraction in AF and moreover RP11‐99E15.2 and RP3‐523K23.2 which could modulate extracellular matrix binding and transcription of HSF2 targets, respectively.^136^ The atrial tissue was also examined in another study considering three AF patients, highlighting 219 differentially expressed lncRNAs.^137^ RNA‐seq performed in lymphocytes of patients with permanent AF versus controls highlighted the differential expression profiles of lncRNAs, ultimately implicating two lncRNAs, ETF1P2 and AP001053.11, in AF pathogenesis.^138^, ^139^ Also focusing on the relevance of lncRNAs as peripheral biomarkers, another study performed a microarray study on blood from patients with AF and matched controls, highlighting 177 deregulated lncRNAs, with the two most deregulated being NONHSAT040387 and NONHSAT098586.^140^ Lastly, a study in atria from AF rabbit highlighted 99,843 putative new lncRNAs, of which TCONS_00075467 was selected to be important for electrical remodeling, possibly through sponging of miR‐328 and subsequent regulation of CACNA1C.^141^ Other lncRNAs implicated in AF include TCONS_00202959,^142^ AK055347,^143^ MIAT,^110^ KCNQ1OT1,^144^ and others extensively reviewed in previous publications.^135^, ^145^, ^146^, ^147^ When focusing on the adipose tissue implication in AF, the number of studies is more restricted, but a very recent work performed a RNA‐sequencing analysis in epicardial adipose tissue samples of patients with persistent non‐valvular AF and sinus rhythm, highlighting 57 differentially expressed lncRNAs.^148^

Numerous lncRNAs have also been found deregulated in CHD, with one recent work highlighting a network of 62 lncRNAs, 332 miRNAs, and 366 mRNA differentially expressed in peripheral blood mononuclear cells (PBMCs) of patients with CHD versus controls.^149^ The screening led to the identification of two lncRNAs, CTA‐384D8.35 and CTB‐114C7.4, as main players in the disease.^149^ Also in this case, an in‐depth classification of both miRNA and lncRNAs involved in CHD was performed by Zhang and colleagues,^150^ which specifically report the implicated lncRNAs to be ANRIL,^151^ H19,^152^ HIF1A‐AS1,^153^ linc‐p21,^154^ RNCR3,^155^ TGFB2‐OT1,^156^ lnc‐Ang362,^157^ HAS2‐AS1,^158^ SMILR,^159^ SENCR,^160^ Meg3,^161^ and lnc‐MKI67IP‐3.^162^

Lastly, lncRNAs are also being investigated for their role in atherosclerotic thrombosis, with multiple recent works focusing especially on this topic.^163^, ^164^, ^165^ These include ANRIL,^166^ LeXis,^167^ RP5‐833A20.1,^168^ MeXis,^169^ and several more, able to act through numerous processes such as vascular remodeling, endothelial dysfunction, leukocyte recruitment, macrophage apoptosis, and cholesterol metabolism.^165^

In conclusion, recent evidence indicates the important roles of lncRNAs in the complex regulatory network of CVD, and many of them could be used as novel therapeutic targets and/or biomarkers for early diagnosis or prognosis for CVD. Indeed, current therapies for CVD such as cardiac hypertrophy currently alleviates symptoms, but new genetic analyses could provide new therapeutic targets.^115^ Modulation of lncRNAs such as Meg3, Plscr4, H19, SNHG1, uc.323, or Ahit could attenuate the increasing size of cardiomyocytes.^117^, ^119^, ^120^, ^121^, ^123^, ^124^ Moreover, a specific class of antisense oligonucleotides, GapmeRs, shows great promise in pharmacological silencing of lncRNAs in vivo,^170^ and even if no clinical trial has been performed, therapeutic GapmeR injections have been found to modulate lncRNAs such as Chast^171^ and Meg3^172^ in animal models of pressure overload or Wisper in myocardial infarction.^108^, ^173^ Moreover, as lncRNAs have been detected in extracellular body fluids, they could be used as biomarkers, and example of this is long intergenic non‐coding RNA predicting cardiac remodeling (LIPCAR), whose plasma levels in humans are associated with left ventricular remodeling after myocardial infarction and with an increased risk of developing heart failure.^174^ Other identified predictors are MIAT,^174^ SENCR,^174^ H19,^174^ NFAT,^175^ MHRT,^175^ ANRIL,^176^ lncPPARδ,^177^and CoroMarker.^178^ Remarkably, four clinical trials are investigating the role of lncRNAs as biomarkers in patients with CVD,^132^ suggesting a strong potentiality for these molecules as disease indicators.

### 4.2 Hypertension

Multiple lncRNAs have been found to be upregulated in the plasma of patients with hypertension, such as AK125261, AK098656, and TUG1.^74^ AK098656, upregulated in hypertensive patients, acts through an increase in proliferation and migration of vascular smooth muscle cells (VSMCs), as it has been shown that it can directly bind to the VSMCs‐specific contractile protein, myosin heavy chain‐11, and an essential component of extracellular matrix, fibronectin‐1, promoting their degradation.^179^ Moreover, AK098656‐overexpressing transgenic rats spontaneously progress to hypertension, presenting increased media thickness and reduced arterial lumen.^180^ The lncRNA TUG1 can also modulate proliferation and migration of rats VSMCs acting as a sponge for miR‐145‐5p and thus inducing the miRNA's target FGF10 and subsequently activating the Wnt/β‐catenin pathway.^181^ Proliferation and migration of VSMCs can also be increased in rats by the lncRNAs XR‐007793 and MRAK048635 P1.^182^, ^183^ Downregulation of MRAK048635 P1 seems to induce VSMCs phenotypic switching from a contractile to a secretory phenotype, representing a potential therapeutic target in the disease.^182^ The lncRNA GAS5 can also modulate PDGF‐induced proliferation and migration of human VSMCs through the sponging of miR‐21, which is indeed able to target platelet‐derived growth factor (PDGF).^184^

A second process that can be modulated by lncRNAs in hypertension is indeed muscular remodeling. Vascular remodeling is an active process that involves changes in cellular growth, apoptosis, migration, inflammation, and production of extracellular matrix proteins. The lncRNA GAS5 can also regulate this process as it can remodel arteries such as the caudal, carotid, renal, and thoracic ones. Indeed, GAS5's knockdown regulate the function of endothelial cells and VSMCs through β‐catenin signaling.^185^ Another previously mentioned lncRNA involved in this process is MALAT1, highly expressed in myocardial and thoracic aortic vascular tissues of hypertensive rats, where it promotes cardiac remodeling through transcriptional repression of MyoD.^186^ The inflammatory process can also be of crucial relevance in the hypertension process. TUG1 also act at this level, as it positively correlates with the expression of inflammatory factors such as PAF, ET‐1, TNF‐α, and hsCRP in the blood serum of hypertensive patients.^187^ Moreover, a novel lncRNA has been named Giver (Growth factor‐ and pro‐Inflammatory cytokine‐induced Vascular cell‐Expressed lncRNA), for its action in modulation of inflammation.^188^ Giver is induced by angiotensin II (AngII) through the recruitment of Nr4a3 to Giver's promoter, and both Giver and NR4a3 were found increased in AngII‐treated human VSMC and in arteries from hypertensive subjects but attenuated in hypertensive patients treated with angiotensin‐converting enzyme inhibitors or angiotensin receptor blockers. It has been hypothesized that Nox1, a gene involved in oxidative stress, may be one of the key effectors through which Giver may promote cell proliferation and inflammation in VSMCs.^188^

Polymorphisms in specific lncRNAs can also induce disease pathology. This is the case of cyclin‐dependent kinase inhibitor 2B antisense RNA 1 (CDKN2B‐AS1), also termed ANRIL and previously mentioned for its implication in CVD, where polymorphisms in its sequence may contribute to higher systolic blood pressure in hypertensive patients.^189^, ^190^ Specifically, it has been found that the SNPs rs10757274, rs2383207, rs10757278, and rs1333049, particularly those within the CDKN2B‐AS1 gene and related haplotypes, may confer an increased susceptibility to hypertension development.^189^

### 4.3 Type 2 Diabetes

At all ages, the risk of T2D rises with increasing body fat. The prevalence of T2D is 3 to 7 times higher in those who are affected by obesity than in normal‐weight adults. Specifically, T2D is an adult‐onset, non‐ insulin‐dependent type of diabetes and is strictly linked to obesity.^75^ In recent years, an increased incidence of T2D among youth is also reported, with obesity and family history of T2D generally present.^191^ Also, in this case, lncRNAs could be crucial players in disease onset and its progression and as this review focuses specifically on obesity‐related metabolic diseases, the next paragraph will highlight potential implications of lncRNAs in T2D.

**FIGURE 2:**
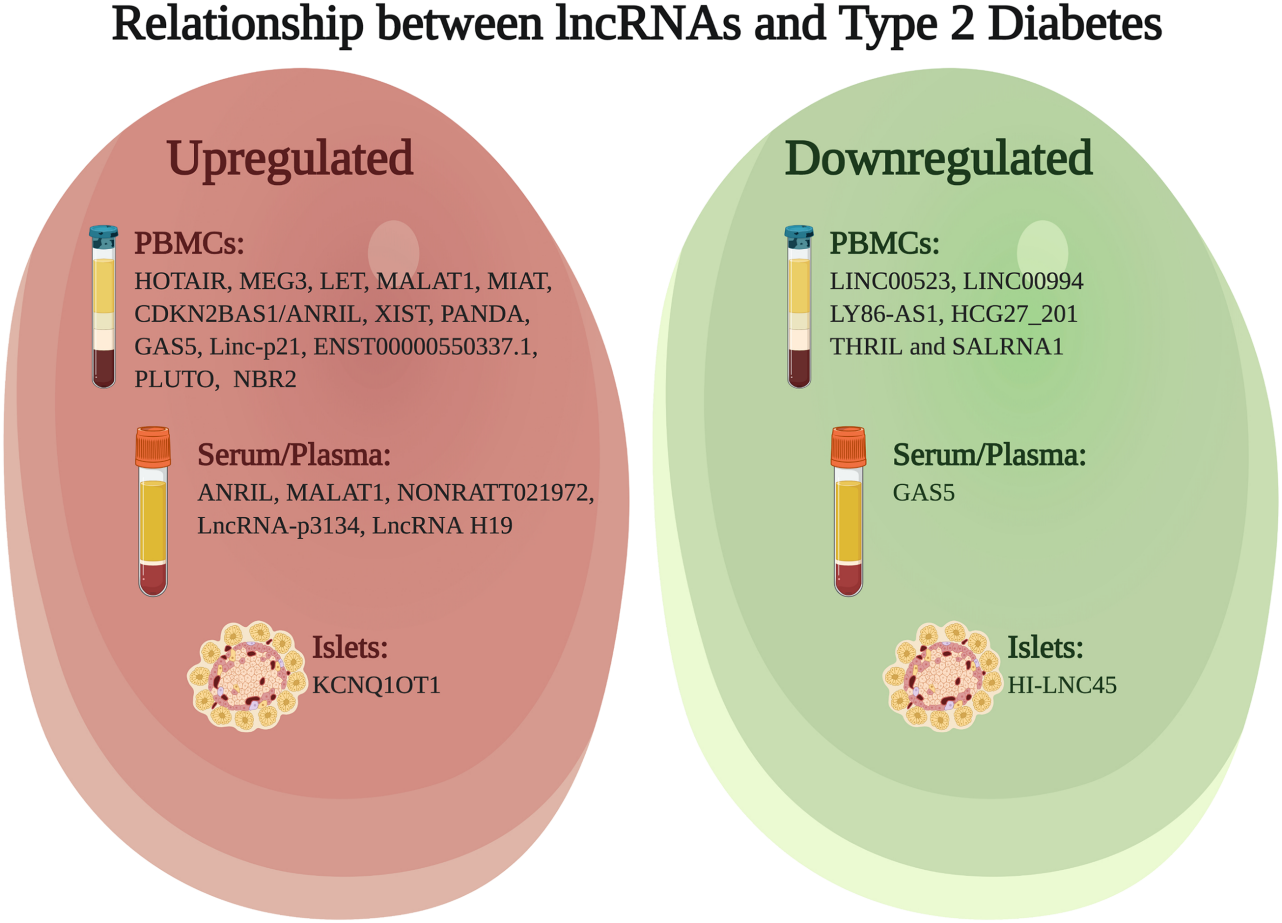
Summary of lncRNAs upregulated or downregulated in specific cell types of patients with T2D. Made in ©BioRender—biorender.com

Indeed, lncRNAs can be both upregulated and downregulated during disease progression in different cell types (Figure 2). Expression profiles of lncRNAs in PBMCs from patients with T2D highlighted how several lncRNAs were significantly increased compared to controls, and these included HOTAIR, Meg3, LET, MALAT1, MIAT, CDKN2BAS1/ANRIL, XIST, PANDA, GAS5, Linc‐p21, ENST00000550337.1, PLUTO, and NBR2.^192^ The lncRNAs ANRIL and MALAT1 were found increased in the serum of patients with T2D,^193^, ^194^ and the same was true for NONRATT021972, which also correlated with increased blood glucose and neuropathic pain.^195^ Interestingly, LncRNA‐p3134 is highly expressed in serum's exosomes of patients with T2D as studies found that it is secreted by islet β‐cell.^196^ Moreover, the lncRNA H19 was found upregulated in plasma of patients with T2D,^197^ and the lncRNA KCNQ1OT1 was upregulated in T2D islets.^198^ Evidences can also be obtained from murine models of the disease, as is the case of E330013P06, which was found upregulated firstly in macrophages of diet‐induced insulin‐resistant T2D mice and subsequently also found upregulated in monocytes from patients with T2D.^199^

Interestingly, many lncRNAs have also been reported to be downregulated in patients with T2D. When considering PBMCs screening studies, results showed that multiple lncRNAs were found downregulated. These include LINC00523, LINC00994,^200^ LY86‐AS1, HCG27_201,^201^ THRIL, and SALRNA1.^192^ Moreover, studies showed that levels of GAS5 lncRNA were decreased both in serum and in plasma of patients with T2D.^197^, ^202^ Lastly, the lncRNA HI‐LNC45 was found downregulated in human T2D islets.^198^

Indeed, lncRNAs can modulate the cellular activity of pancreatic β cells. lncRNA‐p3134, found deregulated in human patients and diabetic mice, seems to act as a new signaling molecule that maintains β‐cell mass and enhances insulin synthesis and secretion, and indeed it has been seen that lncRNA‐p3134 can contribute to reverse the insufficient insulin secretion in T2D.^196^ Moreover, the lncRNA β‐cell long intergenic noncoding RNA (βlinc1) can coordinate the regulation of neighboring islet‐specific transcription factors, and in fact it is necessary for the specification and function of insulin‐producing β cells. In particular, in adult mice it has been shown that deletion of βlinc1 leads to a defective islet development and disruption of glucose homeostasis.^203^ In pediatric age, Liu et al. reported that several lncRNAs involved in regulation of glucose metabolic process and insulin resistance (IR), such as RP11‐559N14.5, RP11‐363E7.4, and RP11‐707P17.1, were significantly upregulated or downregulated in children with obesity compared to controls, even in the absence of diabetes.^31^ Considering that hyperglycemia and T2D develop when the pancreas cannot match the increased insulin demands resulting from IR, the lncRNAs could play a crucial role in the onset of the disease.

### 4.4 Nephropathy

Obesity is a major risk factor for the development of chronic kidney disease, through the direct development of nephropathy.^204^, ^205^, ^206^ Indeed, obesity can cause both a specific renal nephropathy and contribute to renal complications in metabolic syndrome.^206^ LncRNAs have also been found to associate with this process.^207^, ^208^, ^209^ Specifically, the role of lncRNAs in diabetic nephropathy (DN), which accounts for approximately 40% of diagnosed end‐stage kidney failure, has been extensively reviewed by Li and collaborators.^207^ Specifically, TUG1,^210^, ^211^, ^212^ MIAT,^213^ CASC2,^214^ ENSMUST00000147869,^215^ 1700020I14Rik,^216^ CYP4B1‐PS1–001,^217^ Gm15645,^218^ and LINC01619^219^ were downregulated in DN, whereas PVT1, MALAT1,^220^ Gm4419,^221^ Gm15645,^218^ NR_033515,^222^ Erbb4‐IR,^223^ ASncmtRNA‐2,^224^ and lnc‐MCG^225^ were upregulated in DN.^207^

Among the other lncRNAs implicated, Rpph1 was found upregulated in mice with DN, regulating also cell proliferation and inflammatory cytokines production in mesangial cells, through a direct interaction with galectin‐3.^226^ LncRNAs can indeed play a role in epigenetic regulation of DN, along with canonical modulators such as histone modifiers and DNA methylation.^227^ Indeed, they can act synergistically with miRNAs in the disease pathology, as does RP23, which is induced by TGF‐β1 in mesangial cells along with its containing miRNAs, miR‐216a, and miR‐217.^228^ Moreover, in mouse miR‐192 is also co‐regulated by TGF‐β1 in mesangial cells along with its host ncRNA CJ241444, through promoter Smad binding elements and epigenetic regulation via protein C‐ets‐1 and histone acetylation.^227^, ^229^ Lastly, another study found 21 lncRNAs upregulated in two models of renal fibrosis, subsequently downregulated in Smad3‐knockout mice, suggesting they were induced by this factor.^230^

### 4.5 Osteoarthritis

Obesity can impact tissue types other than the adipose tissue, and indeed it can significantly impact both the musculoskeletal and immune systems, leading to the development of OA.^77^, ^231^ OA is a debilitative degenerative joint disorder which is characterized by pain, decreased mobility, and an overall negative impact on the quality of life.^231^ In recent years, lncRNAs have been found to also be strongly deregulated in this disease, although most studies concern OA development and do not specifically focus on the obesogenic co‐morbidity.^77^ These lncRNAs have been extensively reviewed in other works,^77^, ^232^ specifically classifying them for their role in disease progression, immune response, and even potential therapeutic targets.^77^, ^232^ It is indeed clear that the main implication of lncRNAs in OA relates to the immune response, and to this end in recent years mounting studies are reporting this correlation, with the implication of, but not limited to, CASC2,^233^ SNHG1,^234^ DANCR,^235^ HOTAIR,^236^ H19,^237^ SNHG7,^238^ MFI2‐AS1,^239^ PACER, CILinc01, CILinc02,^240^ PVT1,^241^ XIST,^242^ and FOXD2‐AS1.^243^ A high‐throughput screening also reported 3007 upregulated lncRNAs and 1707 downregulated lncRNAs in OA human cartilage compared with normal samples, indicating their significant implication in the diseases.^244^ Moreover, another work investigated the role of exosomal lncRNAs from plasma and from synovial fluid in patients at different stages of OA, highlighting a role for PCGEM1 in disease progression.^245^

Even so, future works will need to specifically focus on the link between OA, lncRNAs, and obesity. Nanus and co‐authors reported 19 differentially expressed lncRNAs in normal‐weight OA versus non‐OA patient fibroblasts, and these are MALAT1, MIR155HG, SMILR, LINC01426, RP11‐863P13.3, CARMN, RP11‐79H23.3, RP11‐362F19.1, RP11‐290 M5.4, VLDLR‐AS1, RP11‐536 K7.3, HAGLR, LINC01915, RP11‐367F23.2, RP11‐392O17.1, LINC01705, LINC01021, DNAJC27‐AS1, and AF131217.1.^246^ Specifically, MALAT1 was rapidly induced upon stimulation of OA synovial fibroblasts with proinflammatory cytokines, and its ablation leads to a reduced expression of IL‐6 and IL‐8.^77^, ^246^ Moreover, the lncRNA Nespas was found upregulated in human OA chondrocytes, sponging numerous miRNAs which target Acyl‐CoA synthetase 6 (ACSL6), leading to an overall increase in ACSL6.^247^ ACSL6 encodes a key enzyme that activates polyunsaturated long‐chain fatty acids, suggesting that this process could modulate lipid metabolism in OA.^247^ Overall, these evidences suggest a clear implication for lncRNAs in mediating epigenetic dysregulation in OA, but the specific link with obesity will need further clarification.

### 4.6 Hepatic metabolic disease

Obesity is also linked with the development of hepatic metabolic disease, as nonalcoholic fatty liver disease (NAFLD) and especially its most severe form (nonalcoholic steatohepatitis) present an increased prevalence in patients with obesity (from 3% to 20–40%).^248^ LncRNAs also appear to intervene in this process, with a tight link with obesity development. Indeed, the lncRNA Blnc1, implicated in adipogenesis and obesity, was found upregulated in obese and NAFLD mice, activating SREBP1c and hepatic lipogenesis, thus aggravating disease progression.^249^ Gm15622 was also found upregulated in the liver of obese mice fed a HFD, exerting its mechanism of action sponging miR‐742‐3p, subsequently upregulating SREBP1c.^250^ Moreover, its inactivation abrogates HFD‐induced hepatic steatosis, suggesting also in this case a therapeutic window.^249^ Conversely, lncARSR was found upregulated in high fatty acid‐treated human HepG2 and NAFLD mouse models, binding YAP1 and further increasing lipid accumulation, a mechanism alleviated when lncARSR was silenced.^251^ The lncRNA H19 was also upregulated in NAFLD murine models, and again its silencing reduced lipid accumulation in hepatocytes.^252^ On the contrary, overexpression of the lncRNA FLRL2 in vivo in murine NAFLD models resolved steatosis, lipogenesis, and inflammation.^253^ Similarly, Meg3 was downregulated in HFD mice, and acting as ceRNA for miR‐21 it could help alleviate lipid over‐deposition.^254^

Also in this case, RNA sequencing and microarrays allowed the identification of numerous new putative candidates. Indeed, numerous high‐throughput studies were performed in both murine models^255^, ^256^, ^257^ and human tissues,^258^ allowing the identification of specific new candidates such as AK012226,^256^ NONMMUT010685,^257^ and MALAT1.^258^ Interestingly, starting from pre‐existing human transcriptome data on NAFLD and liver metabolism, it was also possible to develop a pipeline which identified human lncRNA metabolic regulators (hLMR), with a specific one being strictly involved in cholesterol metabolism.^259^ Their potential as biomarkers was investigated analyzing serum samples of patients with mild and severe NAFLD; through microarray analysis several ncRNAs were identified, and specifically the expression of TGFB2/TGFB2‐OT1 allowed advanced fibrosis discrimination.^260^ Indeed, the amount of data concerning the role of lncRNAs is becoming increasingly overwhelming, with numerous new evidences each year, and for further reading on the topic we refer the reader to other published review reports.^261^, ^262^, ^263^, ^264^, ^265^, ^266^, ^267^

### 4.7 Dyslipidemia

Obesity is probably the main cause for the development dyslipidemia, which typically consists of increased triglycerides, free fatty acids, apolipoprotein B, and LDL‐C, and decreased HDL‐C.^268^ The role of lncRNAs in adipogenesis and thus lipid metabolism has been previously discussed in Section 3, but limited evidence specifically refers to the link between lncRNAs and patients with dyslipidemia.^269^ Among all, Blnc1 activation in epididymal fat in HFD‐induced obese mice seems to have a slight impact on dyslipidemia, suggesting a specific link with this pathogenesis.^270^ Moreover, a recent work screened the lncRNAs expression in rat livers with hypertriglyceridemia and identified the upregulation of a novel lncRNA: lnc19959.2. The knockdown of lnc19959.2 resulted in triglycerides lowering effects both in vitro and in vivo, and mechanistic studies revealed that lnc19959.2 upregulated ApoA4 expression via ubiquitinated transcription inhibitor factor Purb, while its specific interaction with hnRNPA2B1 was able to downregulate the expression of Cpt1a, Tm7sf2, and Gpam.^271^

Indeed, lncRNAs can deeply influence lipid homeostasis, but further studies are required in order to determine whether lncRNAs that regulate lipogenesis, lipolysis, β‐oxidation, adipogenesis, and thermogenesis could also become biomarkers for therapies that target dyslipidemias.^269^, ^272^

## 5 CONCLUSIONS

Obesity is a complex disease representing a great burden on the health care system, commonly leading to the development of co‐morbidities also in pediatrics. Epigenetics through RNA biology might play a crucial role in elucidating new targetable pathways, and in this context lncRNAs are emerging as interesting new candidate targets and players. Indeed, obesity‐associated lncRNAs play a crucial role in adipose tissue modulation, but their action is not limited to this, as they have been implicated in modulating obesogenic co‐morbidities influencing the cardiovascular system, the immune system, the liver, and even the musculoskeletal system.^73^, ^246^, ^265^ Moreover, the number of co‐morbidities associated with obesity is extremely significant and includes also diseases which do not strictly correlate with disruption in metabolic pathways. Indeed, multiple numerous tumors are also obesity‐induced, and although no specific correlation between lncRNAs present in patients with obesity and specific cancer has yet been made, one review report summarizes the link between numerous lncRNAs present both in obesity and cancer.^273^ Non‐coding RNAs will revolutionize modern medicine making it possible to understand in detail unknown aspects of molecular biology over the coming years, and indeed a deep understanding of lncRNAs' role in adipocytes biology will provide multiple novel therapeutic strategies to better combat obesity and prevent early obesity complications in the near future. There is a need to summarize all the recent advances made in the discovery of the role of lncRNAs in the pathogenesis and progression of this disease, and it appears evident that in future years more and more research efforts will focus on characterization of the specificity of lncRNAs' mechanisms of action in obesity‐related diseases (Table 1). Indeed, further studies will need to analyze in depth the transcriptional deregulation present at a tissue level in patients with obesity and co‐morbidities, in order to identify further deregulated targets. A better understanding of these mechanisms, already from pediatric age, will accompany us in filling the gap from basic research to clinical care of patients with obesity. These molecules, in fact, could act as biomarkers for the early diagnosis of obesity‐linked complications and possibly representing new indicators of risk assessment.

**TABLE 1:** Summary of deregulated lncRNAs in obesity and associated diseases

Disease	LncRNA
Obesity	SRA,^67^ ASMER‐1 and ASMER‐2,^68^ RP11‐20G13.3,^31^ PVT1,^50^ Plnc1,^48^ HOTAIR,^72^ lnc19959.2.^271^
Cardiovascular diseases	CAIF,^83^ CDKN2BAS1/ANRIL,^84^, ^151^, ^166^, ^176^ APF,^85^ AK088388,^86^ Meg3,^87^, ^161^ CARL,^88^ MDRL,^89^, ^90^ ZFAS1,^92^, ^93^ HOTAIR,^94^ MALAT1,^95^, ^96^ GAS5,^97^ FAF,^98^ TTTY15,^99^ ECRAR,^100^ AK080084,^101^ NR_045363,^102^ TUG1^103^ and Meg3,^91^, ^104^, ^117^ MIRT1 and MIRT2,^105^ MALAT1,^106^ MHRT,^107^, ^116^, ^175^ Wisper,^108^ MIAT,^109^, ^110^, ^174^ Chaer,^112^ CHRF,^113^ lncR‐UCA1,^114^ DACH1,^118^ H19,^119^, ^152^, ^174^ Plscr4,^120^ SNHG1,^121^ TINCR,^122^ Uc.323,^123^ Ahit,^124^ HRCR,^125^ HypERlnc^126^ RP11‐99E15.2 and RP3‐523 K23.2,^136^ ETF1P2 and AP001053.11,^138^, ^139^ NONHSAT040387 and NONHSAT098586,^140^ TCONS_00075467,^141^ TCONS_00202959,^142^ AK055347,^143^ MIAT,^110^ KCNQ1OT1,^144^ CTA‐384D8.35 and CTB‐114C7.4,^149^ HIF1A‐AS1,^153^ linc‐p21,^154^ RNCR3,^155^ TGFB2‐OT1,^156^ lnc‐Ang362,^157^ HAS2‐AS1,^158^ SMILR,^159^ SENCR,^160^, ^174^ lnc‐MKI67IP‐3,^162^ LeXis,^167^ RP5‐833A20.1,^168^ MeXis,^169^ lncPPARδ^177^ and CoroMarker.^178^
Hypertension	AK125261,^74^ AK098656,^74^, ^179^, ^180^ TUG1,^74^, ^181^, ^187^ XR‐007793,^183^ MRAK048635 P1,^182^ GAS5,^184^, ^185^ MALAT1,^186^ Giver,^188^ CDKN2BAS1/ANRIL.^189^, ^190^
Type 2 diabetes	HOTAIR,^192^ Meg3,^192^ LET,^192^ MIAT,^192^ XIST,^192^ PANDA,^192^ GAS5,^192^, ^197^, ^202^ LINC‐p21,^192^ ENST00000550337.1,^192^ PLUTO,^192^ NBR2,^192^ MALAT1,^192^, ^194^ CDKN2BAS1/ANRIL,^192^, ^193^ NONRATT021972,^195^ LncRNA‐p3134,^196^ H19,^197^ KCNQ1OT1,^198^ E330013P06,^199^ LINC00523,^200^ LINC00994,^200^ LY86‐AS1,^201^ HCG27_201,^201^ THRIL,^192^ SALRNA1,^192^ HI‐LNC45,^198^ lncRNA‐p3134,^196^ βlinc1,^203^ RP11‐559 N14.5,^31^ RP11‐363E7.4,^31^ RP11‐707P17.^31^
Nephropathy	TUG1,^210^, ^211^, ^212^ MIAT,^213^ CASC2,^214^ ENSMUST00000147869,^215^ 1700020I14Rik,^216^ CYP4B1‐PS1–001,^217^ Gm15645,^218^ LINC01619,^219^ PVT1,^274^ MALAT1,^220^ Gm4419,^221^ Gm15645,^218^ NR_033515,^222^ Erbb4‐IR,^223^ ASncmtRNA‐2,^224^ lnc‐MCG,^225^ Rpph1,^226^ RP23,^228^ CJ241444.^227^, ^229^
Osteoarthritis	CASC2,^233^ SNHG1,^234^ DANCR^235^HOTAIR,^236^ H19,^237^ SNHG7,^238^ MFI2‐AS1,^239^ PACER, CILinc01, CILinc02,^240^ PVT1,^241^ XIST,^242^ FOXD2‐AS1,^243^ PCGEM1,^245^ MALAT1,^246^ MIR155HG,^246^ SMILR,^246^ LINC01426,^246^ RP11‐863P13.3,^246^ CARMN,^246^ RP11‐79H23.3,^246^ RP11‐362F19.1,^246^ RP11‐290 M5.4,^246^ VLDLR‐AS1,^246^ RP11‐536 K7.3,^246^ HAGLR,^246^ LINC01915,^246^ RP11‐367F23.2,^246^ RP11‐392O17.1,^246^ LINC01705,^246^ LINC01021,^246^ DNAJC27‐AS1,^246^ AF131217.1,^246^ Nespas.^247^
Hepatic metabolic disease	Blnc1,^249^ Gm15622,^250^ lncARSR,^251^ H19,^252^ FLRL2,^253^ Meg3,^254^ AK012226,^256^ NONMMUT010685,^257^ MALAT1,^258^ hLMR,^259^ TGFB2‐OT1.^260^
Dyslipidemia	Blnc1,^270^ lnc19959.2^271^

## CONFLICT OF INTEREST

The authors declare that they have no conflict of interest.

## Supporting information


**Table S1:** List of lncRNAs cited in the manuscript listed in alphabetical order with the number of known orthologues and a specific focus on their presence in the most common models (*Rattus norvegicus*, *Mus musculus*, and *Homo sapiens*)
