# Supplementary material for: Role of long non‐coding RNAs in adipogenesis: State of the art and implications in obesity and obesity‐associated diseases
Source: Obes Rev. 2021 Jan 14;22(7):e13203. doi: 10.1111/obr.13203 (PMC8244036; doi:10.1111/obr.13203)
Supplement: Supplementary file 1 — Table S1: List of lncRNAs cited in the manuscript listed in alphabetical order with the number of known orthologues and a specific focus on their presence in the most common models (Rattus norvegicus, Mus musculus, and Homo sapiens) [file OBR-22-e13203-s001.pdf]

Role of long non-coding RNAs in adipogenesis: state of the art and implications in obesity and obesity-associated diseases

Federica Rey[1,2], Valentina Urrata[1,2], Luisa Gilardini[3], Simona Bertoli[3,4], Valeria Calcaterra[5,6], Gian Vincenzo Zuccotti[1,2,6], Raffaella Cancello[3], Stephana Carelli[1,2]

[1] Department of Biomedical and Clinical Sciences "L. Sacco", University of Milan, Via Grassi 74, 20157 Milan, Italy

[2] Pediatric Clinical Research Center Fondazione "Romeo ed Enrica Invernizzi", University of Milano, Milano, Italy

[3]Obesity Unit—Laboratory of Nutrition and Obesity Research, Department of Endocrine and Metabolic Diseases, IRCCS Istituto Auxologico Italiano, Milan, Italy

4 International Center for the Assessment of Nutritional Status (ICANS), Department of Food, Environmental and Nutritional Sciences (DeFENS), University of Milan, Milan, Italy

5 Pediatrics and Adolescentology Unit, Department of Internal Medicine, University of Pavia, Pavia, Italy

6 Department of Pediatrics, Children's Hospital "V. Buzzi", Milano, Italy

Correspondence: Dr. Stephana Carelli, Pediatric Research Center "Romeo ed Enrica Invernizzi", Dept. Biomedical and Clinical Sciences, University of Milano, Via G.B. Grassi 74, 20157, MilaNo, Italy. E-mail: stephana.carelli@unimi.it. Tel. 00390250319825

Table S1: List of lncRNAs cited in the manuscript listed in alphabetical order with the number of known orthologues and a specific focus on their presence in the most common models (Rattus Norvegicus, Mus Musculus and Homo Sapiens)

	# of orthologues	Rattus Norvegicus	Mus musculus	Homo Sapiens
1700020I14Rik	5	Yes	Yes	Yes
AC092834.1	N/A	No	No	Yes
ADINR (CEBPA-DT)	N/A	No	No	Yes
AdipoQ AS	N/A	No	Yes	No
ADNCR	N/A (Bos Taurus)	No	No	No
AF131217.1	N/A	No	No	Yes
Ahit	N/A	No	Yes	No
AK012226 (Lrmda)	332	Yes	Yes	Yes
AK055347	N/A	No	No	Yes
AK079912	N/A	No	Yes	No
AK080084	N/A	No	Yes	No
AK088388 (N4bp2)	330	Yes	Yes	Yes
AK098656 (LINC01227)	N/A	No	No	Yes
AK125261 (CDHR3)	256	Yes	Yes	Yes
AP001053.11	N/A	No	No	Yes
APF	N/A	No	Yes	No
ASMER-1	N/A	No	No	Yes
ASMER-2	N/A	No	No	Yes
ASncmtRNA-2	N/A	No	Yes	Yes
Blnc1 (PAQR9)	326	Yes	Yes	Yes
CAIF	N/A	No	No	Yes
CARL	N/A	No	Yes	No
CASC2	N/A	No	No	Yes
CDKN2BAS1/ANRIL	N/A	No	No	Yes
Cebpd	N/A	No	Yes	No
Chaer	N/A	No	No	Yes
CHRF	N/A	No	Yes	No
CILinc01	N/A	No	No	Yes
CILinc02	N/A	No	No	Yes
CJ241444	N/A	No	Yes	No
CoroMarker	N/A	No	No	Yes
CTA-384D8.35	N/A	No	No	Yes
CTB-114C7.4	N/A	No	No	Yes
CYP4B1-PS1-001	N/A	No	Yes	No
DACH1	310	Yes	Yes	Yes
DANCR	5	Yes	Yes	Yes
DNAJC27-AS1	N/A	No	No	Yes
E330013P06 (Carmn)	3	Yes	Yes	Yes
ECRAR	N/A	No	No	Yes
ENSMUST00000147869	N/A	No	Yes	No
ENST00000550337.1 (LINC02402)	N/A	No	No	Yes
Erbb4-IR	N/A	No	Yes	No
ETF1P2	N/A	No	No	Yes

**FAF (FANCF)**	310	Yes	Yes	Yes
**FLRL2**	N/A	No	Yes	No
**FOXD2-AS1**	N/A	No	No	Yes
**GAS5**	3	Yes	Yes	Yes
**Giver**	N/A	No	No	Yes
**Gm15051 (Hotairm1)**	3	Yes	Yes	Yes
**Gm15622**	N/A	No	Yes	No
**Gm15645**	N/A	No	Yes	No
**Gm4419**	N/A	No	Yes	No
**H19**	3	No	Yes	Yes
**HAGLR**	2	No	Yes	Yes
**HAS2-AS1**	N/A	No	No	Yes
**HCG27_201**	N/A	No	No	Yes
**HI-LNC45**	N/A	No	No	Yes
**HIF1A-AS1**	N/A	No	No	Yes
**hLMR**	N/A	No	No	Yes
**HOTAIR**	4	No	Yes	Yes
**HOXA11-AS1**	4	Yes	Yes	Yes
**HRCR**	N/A	No	Yes	No
**HypERlnc**	N/A	No	No	Yes
**IMFNCR**	N/A (Chicken)	No	No	No
**KCNQ1OT1**	N/A	No	No	Yes
**LET**	N/A	No	No	Yes
**LeXis (CT70)**	N/A	No	No	Yes
**Linc-p21**	2	No	Yes	Yes
**LINC00523**	N/A	No	No	Yes
**LINC00994**	N/A	No	No	Yes
**LINC01021 (PURPL)**	N/A	No	No	Yes
**LINC01426**	N/A	No	No	Yes
**LINC01619**	10	No	No	Yes
**LINC01705**	N/A	No	No	Yes
**LINC01915**	N/A	No	No	Yes
**lnc-Ang362 (MIR222HG)**	N/A	No	No	Yes
**lnc-BATE**	N/A	No	Yes	No
**lnc-leptin**	N/A	No	Yes	No
**lnc-MCG**	N/A	No	Yes	No
**lnc-MKI67IP-3 (LINC01826)**	N/A	No	No	Yes
**lnc-ORA**	N/A	No	Yes	No
**lnc19959.2**	N/A	Yes	No	No
**lncARSR**	N/A	No	No	Yes
**lncPPARδ**	N/A	No	No	Yes
**lncR-UCA1**	N/A	No	Yes	No
**lncRNA-Adi**	N/A	Yes	No	No
**LncRNA-p3134**	N/A	No	Yes	Yes
**LY86-AS1**	N/A	No	No	Yes
**MALAT1**	2	No	Yes	Yes
**MDRL**	N/A	No	Yes	No
**MEG3**	2	No	Yes	Yes
**MeXis**	N/A	No	Yes	No

MFI2-AS1	N/A	No	No	Yes
MHRT	N/A	No	No	Yes
MIAT	3	Yes	Yes	Yes
MIR31HG	2	No	No	Yes
MIR155HG	6	Yes	Yes	Yes
MIR221HG	N/A	No	No	Yes
MIRT1	N/A	No	Yes	No
MIRT2	N/A	No	Yes	No
MRAK048635 P1	N/A	Yes	No	No
NEAT1	2	No	Yes	Yes
NBR2	N/A	No	No	Yes
Nespas (GNAS-AS1)	N/A	No	No	Yes
NONHSAT040387	N/A	No	No	Yes
NONHSAT098586	N/A	No	No	Yes
NONMMUT010685	N/A	No	Yes	No
NONRATT021972	N/A	Yes	No	Yes
NR_033515 (Cyp2d37-ps)	N/A	No	Yes	No
NR_045363 (2810429I04Rik)	N/A	No	Yes	No
PACER	N/A	No	No	Yes
PANDA	N/A	No	No	Yes
PCGEM1	N/A	No	No	Yes
Plnc1	N/A	No	Yes	No
Plscr4	140	Yes	Yes	Yes
PLUTO	N/A	No	No	Yes
PVT1	2	No	Yes	Yes
PU.1 AS	N/A	No	Yes	No
RNCR3 (MIR124-1HG)	2	No	Yes	Yes
RP11-20G13.3 (LINC02244)	N/A	No	No	Yes
RP11-290M5.4	N/A	No	No	Yes
RP11-362F19.1	N/A	No	No	Yes
RP11-363E7.4	N/A	No	No	Yes
RP11-367F23.2	N/A	No	No	Yes
RP11-392O17.1	N/A	No	No	Yes
RP11-536K7.3	N/A	No	No	Yes
RP11-559N14.5	N/A	No	No	Yes
RP11-707P17	N/A	No	No	Yes
RP11-79H23.3	N/A	No	No	Yes
RP11-863P13.3	N/A	No	No	Yes
RP11-99E15.2	N/A	No	No	Yes
RP23 (OFD1)	393	Yes	Yes	Yes
RP3-523K23.2	N/A	No	No	Yes
RP5-833A20.1	N/A	No	No	Yes
Rpph1	N/A	No	No	Yes
SALRNA1	N/A	No	No	Yes
SENCR	N/A	No	No	Yes
slincRAD	N/A	No	Yes	No
SMILR	N/A	No	No	Yes
SNHG1	N/A	No	No	Yes
SNHG7	N/A	No	No	Yes

**SRA**	289	Yes	Yes	Yes
**TCONS_00075467**	N/A (Rabbit)	No	No	No
**TCONS_00202959**	N/A	Yes	No	No
**TGFB2-OT1**	N/A	No	No	Yes
**THRIL**	N/A	No	No	Yes
**TINCR**	3	Yes	Yes	Yes
**Tmem189**	176	No	Yes	Yes
**TTTY15**	N/A	No	No	Yes
**TUG1**	4	Yes	Yes	Yes
**Uc.323**	N/A	No	Yes	No
**VLDLR-AS1**	N/A	No	No	Yes
**Wisper**	N/A	No	Yes	Yes
**XIST**	N/A	No	No	Yes
**XR-007793**	N/A	Yes	No	No
**ZFAS1**	N/A	No	No	Yes
